# Supplemental Nutrition Assistance Program participation and health care expenditures in children

**DOI:** 10.1186/s12887-022-03188-3

**Published:** 2022-03-24

**Authors:** Stephen Rogers, Arvin Garg, Yorghos Tripodis, Annelise Brochier, Emily Messmer, Mikayla Gordon Wexler, Alon Peltz

**Affiliations:** 1 Department of Pediatrics, Boston University School of Medicine, Boston Medical Center, 801 Albany St 2nd Floor, Boston, MA 02119 USA; 2 Present address is Children’s Hospital of Philadelphia, 3401 Civic Center Boulevard, Philadelphia, PA 19104 USA; 3 Department of Pediatrics, University of Massachusetts Medical School, 55 Lake Avenue North, Worcester, MA 01655 USA; 4 Department of Biostatistics, Boston University School of Public Health, Crosstown Center, 801 Massachusetts Ave, Boston, MA 02118 USA; 5 Department of Pediatrics, Boston Medical Center, 801 Albany St 2nd Floor, Boston, MA 02119 USA; 6 Department of Population Medicine, Harvard Medical School, Harvard Pilgrim Health Care Institute, 401 Park Drive, Suite 401 East, Boston, MA USA

**Keywords:** SNAP, Health care expenditures, Medical Expenditure Panel Survey

## Abstract

**Background:**

The Supplemental Nutrition Assistance Program (SNAP) has well-established positive impacts on child health outcomes, including increased birth weight and decreased likelihood of underweight status. Studies in adult populations suggest that SNAP is associated with lower health care costs, although less is known in children.

**Methods:**

Retrospective analysis of U.S. children (age <18 years) living in low-income households (< 200% of the federal poverty level) in the 2013-2017 Medical Expenditure Panel Survey. We used multivariable regression, adjusting for sociodemographic and clinical covariates, to model the effect of continuous SNAP enrollment on health expenditures as compared to non-enrollees at 12 and 24 months.

**Results:**

The sample included 5,626 children, of whom 49.2% consistently received SNAP for the entire two-year survey period. Compared with SNAP non-recipients, SNAP-recipient households more often had incomes below 100% FPL (78.3% vs 37.9%), and children in SNAP-recipient households were more often publicly insured (94.9% vs 64.5%). Unadjusted expenditures were lower for children in SNAP-recipient households at 12 ($1222 vs $1603) and 24 months ($2447 vs $3009). However, when adjusting for sociodemographic and clinical differences, no statistically significant differences in health care expenditures, including emergency department, inpatient, outpatient, and prescription costs, were identified.

**Conclusion:**

SNAP participant children experience heightened social hardships across multiple domains. There were no differences in short term health care costs based on SNAP enrollment when accounting for differences in sociodemographic and clinical factors. Despite demonstrated child health benefits, we found that sustained enrollment in SNAP over a two-year period did not generate significant short- term health care cost reductions. Our findings suggest that although SNAP is intended to act as a benefit towards the health and well-being of its recipients, unlike among adults, it may not reduce health care costs among children.

## Introduction

Approximately one in five children in the United States experiences food insecurity, meaning that their access to adequate food is limited by a lack of money or other resources in the household [1]. The COVID-19 pandemic further exacerbated household food insecurity, as significantly more families experienced periods of unemployment or underemployment, with associated financial hardships and an inability to meet basic needs [2]. It is well-established that food insecurity can have deleterious short- and long-term effects on children’s health and development, including increased odds of being hospitalized, increased risk of asthma and other chronic health conditions, and lower test scores and behavioral outcomes in school [3–7].

The Supplemental Nutrition Assistance Program (SNAP) is an important federal food assistance program that assists many low-income families in becoming and remaining food-secure [8–12]. Approximately one-quarter of all US children receive SNAP supports, representing 17 million households of SNAP recipients [13, 14]. Prior studies have demonstrated the positive effects of SNAP for both adults and children through subjective measures, such as associations with better self-reported health status in recipients, and objective measures, including increased birth weight and decreased likelihood of underweight status among children in recipient households [12, 15, 16]. Reduced healthcare expenditures in SNAP recipients for adults are likely a result of improved overall health related to better nutrition. For example, in those with diabetes, proposed mechanisms linking SNAP receipt and improved health include increased fruit and vegetable intake and better glycemic control [17]. While not the original intent of SNAP, the program’s measurable effects on the health of its recipients, which go beyond just alleviating hunger, raise the question of whether a corresponding effect on health care expenditures also occurs. A prior study demonstrated that receipt of SNAP in low-income adults is associated with lower health care expenditures over the course of 24 months when compared with eligible non-recipients [18]. However, to date, it remains unknown whether this association holds true among children. Investigating this association is important because it carries significant policy and clinical practice implications. For example, findings could provide evidence supporting cost-saving, innovative partnerships between social services and medical homes, and for novel pediatric care delivery models that address food insecurity [19]. Some insurers and state Medicaid agencies have already begun to implement direct nutrition supports under value based care arrangements, with the explicit goal of reducing costs; the relationship between SNAP, as a robust pre-existing food insecurity intervention, and health care costs outside the adult population is deserving of further investigation [20, 21]. Thus, in this study we used data from a nationally representative survey to compare longitudinal health care expenditures among children in low-income households who received SNAP to those of children who did not receive SNAP, while accounting for sociodemographic and clinical differences in program enrollment.

## Methods

### Data Source

We used the Medical Expenditure Panel Survey (MEPS) longitudinal data files from the years 2013 through 2017 [22]. MEPS is a nationally-representative survey of U.S. households conducted annually by the Agency for Health Care Research and Quality, focused primarily on insurance and health care expenditures. Households selected to participate in the survey are drawn from respondents to the National Health Interview Survey, and data from each family are collected for two calendar years, over the course of five rounds of interviews [23]. The survey is administered using computer-assisted interviews with response rates between 43.8% and 54.7% for the panels we included in this study.

### Study population

We identified a study population of children (age <18 years) residing in low-income households, defined by a household income <200% of Federal Poverty Level (FPL). This income level was selected to correspond with the upper limit for broad-based categorical eligibility rules employed in a majority of states, and to align with definitions used in prior studies of SNAP enrollment on health outcomes [18, 23, 24]. As the objective of the study was to examine impacts on health care costs, we excluded children who did not have any health care encounters during the entire study period (7.3% of the sample). There were similar numbers of SNAP and non-SNAP enrollees among those with zero annual health expenditures. We excluded those with zero health care expenditures from the study because it would have been difficult to determine whether or not SNAP enrollment had any impact on their health care utilization; focusing on children with non-zero health care expenditures allows us to better isolate the relationship between SNAP receipt and healthcare expenditures.

### Medical and sociodemographic covariates

We *a priori* defined a set of child- and household-level sociodemographic, social, and clinical covariates shown to predict both SNAP enrollment and health care expenditures [18, 25]. We used the conceptual framework established by the National Academies of Science, Engineering, and Medicine to identify social risk factors shown to impact health-related outcomes, and also based on the covariates selected in similar studies using MEPS data [3, 25, 26]. Child-level covariates included age, sex, race, ethnicity, insurance coverage, and special health care needs status. MEPS identifies children with special health care needs (CSHCN) using questions adapted from the CSHCN Survey developed by the Child and Adolescent Health Measurement Initiative; these questions assess for whether or not the child uses prescribed medications, is limited in or prevented from doing things that most other children of the same age can do, or receives special therapy [27]. Information on race and ethnicity was self-reported, with imputation conducted by MEPS using other family members for missing/incomplete responses. Household-level covariates included census region, household size, household income relative to FPL, language the survey was conducted in, and the number of parents identified in the household. The parent-level covariates of parental education level of less than high school completed, unemployment, and age were also included.

### Continuous SNAP Enrollment

We defined receipt of SNAP benefits as self-report of receiving continuous SNAP benefits for 24 consecutive months. We estimated SNAP enrollment based on a single question which retrospectively ascertains if anyone in the household received benefits from the Supplemental Nutrition Assistance Program (SNAP) during the calendar year. This question is included in the final survey administration of each calendar year in the sample. We compared this group to children with no SNAP enrollment at all during the study period. We selected this comparison because it represented the most conservative approach for isolating the sustained longer-term effect of SNAP supports. Children in households who did not receive SNAP benefits the entire study period (e.g., received between 1 and 23 months of SNAP benefits) were excluded from the analysis to better isolate the relationship between SNAP receipt and health care expenditures. SNAP receipt in the survey was defined at the level of the household.

### Outcomes

The primary outcome measure was annual health care expenditures expressed in 2017 US dollars, adjusted for inflation using the gross domestic product price index [28]. Health care expenditures recorded in MEPS are equal to the sum of all direct payments for care provided during the survey period, which include out-of-pocket payments and payments made by Medicaid, Medicare, and privates insurers. This data is self-reported, and MEPS performs a supplementary survey of health care providers to validate the data that is collected from households [29]. Expenditure data that is missing from MEPS is imputed during database development using the “hot-deck” method, which matches events which are missing data with events that contain data and are similar on a range of predictors, selected based on the imputed value in question; no further edits to the data were made during this study [30]. We further stratified health expenditures into specific health care settings and domains, including the emergency department (ED), inpatient hospitalizations, outpatient hospital and non-hospital visits, and prescription medications.

### Statistical analysis

We first conducted descriptive statistics of the study cohort to compare sociodemographic and clinical differences between those with continuous SNAP receipt and those without SNAP receipt. Next, we considered several different statistical modeling approaches for accounting for potential selection effects caused by families with higher levels of unmeasured social and/or medical need receiving SNAP. We did this because although we included a large number of covariates, it is recognized that unmet social needs of families often cluster, and we were not able to account for all potential factors in the model. The base model for the study was a linear regression with sample weight. The analysis was limited to the sub-population of children meeting the inclusion criteria, while retaining the entire sample to preserve the complex survey design.

Each linear regression model included all the previously listed covariates, including child- and parent-level sociodemographic characteristics and special health care need status in children with sampling weights and strata from MEPS. We used causal inference techniques to develop two additional models: Sensitive Model A: 1:1 propensity score matching based on sociodemographic characteristics and Sensitive Model B: a linear model with inverse probability weighting [9]. The 1:1 matching used the propensity scores for SNAP receipt from all the previously listed covariates with a greedy algorithm and a caliper of 0.25. We used the propensity scores to calculate inverse probability weights, which we used in a linear model with SNAP receipt as the only predictor [31]. All analysis was performed in SAS, version 9.4.

## Results

### Study population

**Fig. 1:**
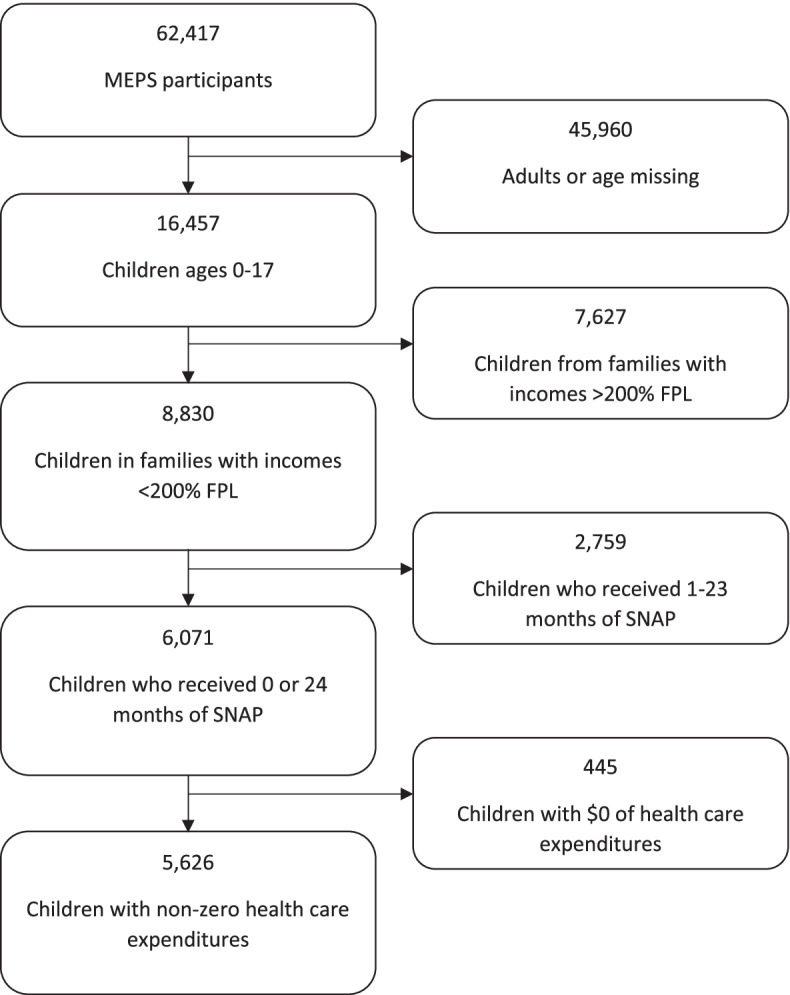
Selection of the final population from the complete sample of Medical Expenditure Panel Survey respondents. Legend: “Selection of the final population from the complete sample of Medical Expenditure Panel Survey respondents.”

The final sample included 5,626 children in low-income households, of whom 2,769 (49.2%) lived in households which reported continuous SNAP benefits for 24 consecutive months during the study period (Fig. 1).

### Sociodemographic characteristics of children in SNAP-recipient households

Among children included in our analysis (<200% FPL, with SNAP for 0 or 24 months), race/ethnicity, region, insurance coverage, presence of special health care needs, parental education level, and parental report of poor/fair mental health were all correlated with whether or not any medical expenditures were reported during the study period. The largest difference in likelihood of expenditures being reported was based on insurance coverage, in which only 71.7% of uninsured children had medical expenses reported, compared to 94.6% for those with public insurance or 94.5% with any private insurance (chi-square <0.0001). Compared to low-income children who did not live in a household receiving SNAP, children receiving SNAP were more likely to be non-Hispanic Black (29.4 vs 14.0%), to have special health care needs (33.3 vs 21.9%), and to be covered by public insurance only (94.9 vs 64.5%, *P*<.001 for all). SNAP recipient households with children were more likely to have an income under 100% FPL (78.3 vs 37.9%), to be headed by a single parent (58.8 vs 30.1%), and to have no parents living in the household who have completed high school (30.0 vs 15.7%, *P*<.001 for all). Parents in SNAP-recipient households were more likely to report fair or poor mental health status (13.0 vs 6.9%, *P*<.001) (Table 1).

**Table 1:** Sociodemographic characteristics of children in SNAP recipient and SNAP non-recipient households

Sociodemographic characteristics	SNAP non-recipients (*N*=2857)	SNAP recipients (*N*=2769)	*P* value
Child age, mean (95% CI)	9.1 (8.8, 9.4)	8.3 (8.0, 8.6)	<.001
Male sex, No. (%)	1477 (50.7)	1406 (50.2)	.70
Race and ethnicity, No. (%)			<.001
Hispanic	1672 (40.1)	1266 (33.2)	
Non-Hispanic Black	430 (14.0)	938 (29.4)	
Non-Hispanic White	541 (33.8)	384 (26.9)	
Other	273 (11.3)	204 (9.6)	
Non-English primary language, No. (%)	1202 (25.5)	825 (20.3)	.07
Census region, No. (%)			.04
Midwest	445 (20.6)	470 (20.3)	
Northeast	337 (13.3)	466 (16.8)	
South	1066 (37.7)	1279 (43.6)	
West	1009 (28.4)	554 (19.3)	
Children with special health care needs, No. (%)	573 (21.9)	855 (33.3)	<.001
Income level, No. (%)			<.001
<100% FPL	1207 (37.9)	2221 (78.3)	
100%-149% FPL	894 (17.7)	403 (16.8)	
150%-199% FPL	719 (30.8)	131 (5.0)	
Insurance coverage, No. (%)			<.001
Any private	629 (29.8)	85 (4.0)	
Public only	2058 (64.5)	2642 (94.9)	
Uninsured	170 (5.7)	42 (1.1)	
Household size, mean (95% CI)	4.5 (4.3, 4.6)	4.6 (4.4, 4.8)	<.001
Parental age, mean (95% CI)	34.9 (34.3, 35.6)	33.1 (32.7, 33.6)	<.001
Parental education level less than high school completed, No. (%)	573 (15.7)	806 (30.0)	<.001
Parental unemployment, No. (%)	60 (2.4)	103 (5.2)	<.001
Parental fair/poor mental health, No. (%)	189 (6.9)	346 (13.0)	<.001
Single-parent household, No. (%)	812 (30.1)	1650 (58.8)	<.001

*SNAP* Supplemental nutrition assistance program

### Health expenditures

Compared with SNAP non-recipient children, continuous SNAP-recipient children had lower *unadjusted* health care expenditures over 12- ($1222 vs $1603; unadjusted difference: -$381; *P* < .001) and 24-month periods ($2447 vs $3009; unadjusted difference -$562; *P* < .001). However, after adjusting for child-, parent-, and household-level covariates, multivariable regression modeling did not demonstrate a statistically significant difference in health care expenditures between SNAP recipients and non-recipients over the course of the first year ($1997 vs $2256; adjusted difference: -$259, 95% confidence interval [CI]: -$645 to $127; *P* =.19) or over 24 months ($3538 vs $4275; adjusted difference: -$737, 95% CI: -$1493 to $18.66; *P* = .06). Receipt of SNAP was not associated with a statistically significant difference in emergency department expenditures (over 24 months: $167.27 vs $171.28; adjusted difference: -$4.00, 95% CI: -$49.61 to $41.60; *P*=0.86), inpatient hospital expenditures (over 24 months: $1434.42 vs $1856.62; adjusted difference: -$422.20, 95% CI: -$986.43 to $142.04; *P*=0.14), outpatient hospital-based expenditures (over 24 months: $178.63 vs $184.51; adjusted difference: -$5.88, 95% CI -$114.23 to $102.48; *P*=0.91), outpatient office-based expenditures (over 24 months: $747.94 vs $889.08; adjusted difference -$141.14, 95% -$297.79 to $15.50; *P*=0.08), or prescription medication expenditures (over 24 months: $622.42 vs $643.10; adjusted difference -$20.68, 95% CI -$198.37 to $157.02; *P*=0.82) (Table 2). Analysis using 1:1 matching and inverse probability weighting produced consistent results, with no statistically significant differences in expenditures or between SNAP recipients and non-recipients. Most children had no emergency department (76.8%) or inpatient hospital (95.8%) expenditures.

**Table 2:** Differences in Health Care Expenditures Between SNAP Recipients and non-Recipients, Adjusted for Sociodemographic Characteristics

	12 months		24 months	
	Mean Difference (95% CI)	***P*** value	Mean Difference (95% CI)	***P*** value
Mean health care expenditures, $	-259.03 (-645.24 to 127.18)	.19	-737.05 (-1492.77 to 18.66)	.06
Emergency department	-6.89 (-32.32 to 18.55)	.59	-4.01 (-49.61 to 41.60)	.86
Expenditures, $				
Inpatient hospital	-186.87 (-482.15 to 108.41)	.21	-422.20 (-986.43 to 142.04)	.14
Expenditures, $				
Outpatient hospital	17.11 (-64.96 to 99.17)	.68	-5.88 (-114.23 to 102.48)	.91
Expenditures, $				
Outpatient non-hospital	-6.28 (-89.80 to 77.23)	.88	-141.14 (-297.79 to 15.50)	.08
Expenditures, $				
Prescription medication	-6.06 (-105.85 to 93.73)	.90	-20.68 (-198.37 to 157.02)	.82
Expenditures, $				

*SNAP* Supplemental nutrition assistance program

The multivariable model used to generate this table included all covariates from Table 1

## Discussion

In this nationally representative sample of U.S. children, we found no statistically significant association between receipt of SNAP and lower health care expenditures (total, emergency department, inpatient, outpatient, prescription costs) when accounting for child-, parent-, and household-level sociodemographic and medical covariates. Consistent with previous studies, we found that SNAP participation is concentrated among families with high levels of unmet medical and social needs, limited parental educational attainment, and high rates of public insurance; the degree of vulnerability of SNAP-recipient families underlines the importance of this program, regardless of cost-saving considerations.

Our results differ from those of recent studies in adult SNAP recipients, which have found that SNAP was associated with lower health care expenditures [8, 18]. One potential reason for this discrepancy between adult and child health care expenditures may be that the specific health conditions most impacted by receipt of SNAP in children might exert their effects on health care expenditures over a longer time period than was studied here. Whereas for adults, high prevalence conditions, such diabetes, may be more immediately impacted by food security and manifest in a more acute need for health care services [17]. We studied cumulative expenditures over 24 months (the longest interval available in this dataset) and did note a trend towards significance (P=0.06), which supports this suggestion. Further, many studies of SNAP’s effects on child health focus on nutrition-related conditions such as low birth weight and childhood obesity, both of which have been found to be associated with significant lifetime costs [32, 33]. The results of this study suggest that longer time horizons, likely well beyond 24 months, are needed to detect the effects that programs which moderate food insecurity have on health care expenditures. It is also worth noting that per-person child health care expenditures are lower than expenditures for adults ($2,479 per child in 2018, $5,644 per adult in 2018 based on MEPS data), so at baseline there may be less potential for short-term cost saving at baseline in children compared to adults.

Although we note that on an unadjusted basis, SNAP participants experience lower health care costs, this difference is not statistically significant when accounting for sociodemographic and clinical differences between the groups in the adjusted model. This finding suggests that unadjusted differences in health care expenditures likely relate, in large part, to underlying sociodemographic and clinical differences between continuous SNAP enrollees and non-SNAP participants, and not to participation in the program itself. We attempted to include as comprehensive a set of covariates as was possible with variables included in the survey, and found that SNAP recipients had significantly more social vulnerabilities than non-recipients, indicating that there may be other unmeasured covariates present. Recognizing that we could not comprehensively account for all co-factors, we also employed two statistical approaches aimed at accounting, to some degree, for unmeasured selection effects. Both approaches showed similar, non-significant effects of program enrollment on outcomes.

The results of this study carry implications for public policy pertaining to SNAP and other nutrition assistance programs. In recent years there has been vigorous political debate regarding the future of federal and state SNAP supports. On an unadjusted basis, policymakers may note lower (unadjusted) health care costs among SNAP participants, but our analysis suggests this may be driven primarily by the fact that SNAP participants often experience worse health and greater unmet social needs, increasing their risk for higher health care costs. When accounting for these differences, we note that over 24 months, unlike adults, children do not appear to have lower health care costs when enrolled in SNAP. It is critically important to note that the intended interpretation of this study is not to suggest a lack of program efficacy. The health and development benefits of SNAP participation for low-income children have been well-established, including fewer days of missed school, improved overall reported child health, fewer asthma-related ED visits, and improved birth outcomes, and these findings do not refute those points [8, 16, 34–36]. Instead, we apply a health care cost framework onto SNAP participation, as a reflection of the growing movement towards the integration of social care into health care delivery. Better recognizing food insecurity and facilitating referrals to SNAP are immensely valuable functions for child health. Our results further reinforce the vulnerability of SNAP participants across other social domains, and suggest that expectations for moderating food insecurity to translate into immediate reduction in health care expenditures may be misplaced. We thus caution against misapplication of a health care financial model on a public health program which was not envisioned as a source of health care cost-savings, but instead as a societal and public health investment towards improving children’s nutrition, health, and well-being. It is our hope that these results might allow policymakers to manage their expectations on the financial returns of the program, and to encourage further research into the unique mechanisms through which SNAP affects health in children compared to adults.

Limitations of this study include the largely self-reported nature of the data, as well as the use of imputed data to replace missing values in MEPS. The accuracy of self-reported data in MEPS may vary; for example, a validation study found that MEPS households were accurate in their reports of inpatient admissions, but underreported ED visits by one-third [37]. Restricting our analysis to children limited this study’s sample size relative to the entire population of MEPS participants, most of whom are adults. Our definition of receipt of SNAP benefits (24 consecutive months of benefits) further limited the sample size by excluding children who received SNAP benefits over a shorter time period from the analysis; however, because we detected no statistically significant difference in health care expenditures when using our definition of SNAP receipt, there is little reason to hypothesize that shorter-term exposure to SNAP would have a different effect. The version of the MEPS datasets used in this study did not include state-level identifiers, precluding us from using this information when adjusting for covariates. Finally, the longest longitudinal period used in this study was two years, and it is possible that the effects of SNAP on overall health and ensuing health expenditures in children could take longer than this relatively short time period to become apparent.

## Conclusions

Using a national sample of low-income U.S. children, we found no significant differences in short-term health care expenditures for continuous SNAP recipients as compared to those who did not enroll in the program. Previous evidence highlights nutritional and development benefits of SNAP, and our results suggest tempering expectations for short-term health care associated cost-savings with SNAP participation in children.

## Data Availability

All datasets analyzed during the current study are available through the Medical Expenditure Panel Survey (https://www.meps.ahrq.gov/mepsweb/data_stats/download_data_files.jsp). All data are publicly available, and public access to this database is open. All datasets are de-identified.

## Abbreviations

SNAP: Supplemental Nutrition Assistance Program
MEPS: Medical Expenditure Panel Survey
FPL: Federal Poverty Level
